# Tuning the Emission Energy of Chemically Doped Graphene Quantum Dots

**DOI:** 10.3390/nano6110198

**Published:** 2016-11-03

**Authors:** Noor-Ul-Ain, Martin O. Eriksson, Susann Schmidt, M. Asghar, Pin-Cheng Lin, Per Olof Holtz, Mikael Syväjärvi, G. Reza Yazdi

**Affiliations:** 1 Department of Physics, The Islamia University of Bahawalpur, 63100 Bahawalpur, Pakistan; mhashmi@iub.edu.pk; 2 Department of Physics, Chemistry and Biology, Linköping University, SE-58183 Linköping, Sweden; marer@ifm.liu.se (M.O.E.); sussc@ifm.liu.se (S.S.); pinli332@student.liu.se (P.-C.L.); poh@ifm.liu.se (P.O.H.); misyv@ifm.liu.se (M.S.)

**Keywords:** graphene quantum dots, emission energy, XPS, photoluminescence, time resolved photoluminescence

## Abstract

Tuning the emission energy of graphene quantum dots (GQDs) and understanding the reason of tunability is essential for the GOD function in optoelectronic devices. Besides material-based challenges, the way to realize chemical doping and band gap tuning also pose a serious challenge. In this study, we tuned the emission energy of GQDs by substitutional doping using chlorine, nitrogen, boron, sodium, and potassium dopants in solution form. Photoluminescence data obtained from (Cl- and N-doped) GQDs and (B-, Na-, and K-doped) GQDs, respectively exhibited red- and blue-shift with respect to the photoluminescence of the undoped GQDs. X-ray photoemission spectroscopy (XPS) revealed that oxygen functional groups were attached to GQDs. We qualitatively correlate red-shift of the photoluminescence with the oxygen functional groups using literature references which demonstrates that more oxygen containing groups leads to the formation of more defect states and is the reason of observed red-shift of luminescence in GQDs. Further on, time resolved photoluminescence measurements of Cl- and N-GQDs demonstrated that Cl substitution in GQDs has effective role in radiative transition whereas in N-GQDs leads to photoluminescence (PL) quenching with non-radiative transition to ground state. Presumably oxidation or reduction processes cause a change of effective size and the bandgap.

## 1. Introduction

Graphene, a two-dimensional honeycomb lattice of sp^2^-bonded carbon atoms, has zero band gap which limits its application in photonics and optoelectronics. The main approaches for band gap engineering are doping and surface modification. This may be obtained by further reducing the lateral dimensions of graphene into nanoribbons and quantum dots (QDs) [1,2,3]. Accordingly, graphene quantum dots (GQDs), which are graphene sheets with -dimensions less than 30 nm, have attracted much interest in recent research due to their unique properties like low toxicity [4], water solubility, biocompatibility [5], and cost effective synthesis. The additional quantum confinement in GQDs creates a band gap and gives rise to photoluminescence (PL) [6,7,8,9]. Lu et al. and Ritter et al. demonstrated using scanning tunneling spectroscopy experiments that the band gap of GQDs directly depends upon their size [10,11]. The band gap modification provides a red- or blue-shift in the PL spectra. According to the first principle theory, the band gap of a nanostructure (hereafter, nanostructure will be referred to as GQDs) increases as its size decreases and leads to a blue-shift of its PL [12,13]. The PL of GQDs is one of their most outstanding features since it can be tuned from blue to red by modifying their size, surface functionalization, edge structure, and chemical doping.

Chemical functionalization and heteroatom doping in GQDs are the common methods of band gap modification also used to modulate their electronic and chemical properties. In heteroatom doping of GQDs, substitutional doping is more effective than the interstitial doping. The heteroatom doping strongly changes the band structure and makes them useful for applications such as gas sensors, bio-imaging, energy converters, photodetectors, solar cells, and LEDs [13,14]. Although substitutional doping may be done by several methods, solution phase approaches are rarely reported in the literature [15]. However, solution phase fabrication has some advantages. Zhu et al. synthesized GQDs with controllable surface chemistry by oxidation using hydrochloric acid (HCl) and reduction using sodium borohydride (NaBH_4_) in GQDs [16]. They have shown multicolor fluorescence and up-conversion in PL. Lin et al. reported the enhanced PL by following reduction of GQDs using NaBH_4_. They proposed that the reduced GQDs serve as fluorescent probe for biological applications [17]. Jin et al. demonstrated tuning of PL by functionalizing GQDs by alkyl amine groups after their fabrication from graphene oxides [13]. They showed that charge transfer takes place between amine functional groups and GQDs which changes the band gap and results in a red-shift of the PL. Li et al. adopted an electrochemical route for preparation of GQDs with green luminescence and explained that the oxygen containing groups facilitate functionalization of GQDs for various optoelectronic devices [18]. Yeh et al. reported the hydrothermal cutting of graphene sheets into GQDs under acidic and alkyl treatment and the resultant blue luminescence [19].

With these observations of tunability of the band gap of GQDs, the efforts initiated a first understanding of this phenomenon. Gupta et al. demonstrated that by functionalizing GQDs, charge transfer takes place between functional groups and the GQDs, which changes the electron density and thereby affects the PL [20]. It has been shown that the blue-shift of the PL peaks is due to the widening of the band gap, whereas the red-shift is caused by the narrowing of the band gap [13]. It is also reported that the observed shift in PL is because of emission from defects states and intrinsic states [21]. Generally, intrinsic state emission is ascribed to the quantum size effect and recombination of localized electron-hole pairs [7,21], whereas defects states arise from structural disorder and oxygen functional groups—i.e., epoxy, hydroxyl, carboxyl and carbonyl groups [22]. These defects are helpful for dissociation of GQDs in water and also they interact widely with organic and inorganic materials which widen the range of potential applications of the GQDs. These defects can be controlled by changing the amount of oxygen containing groups—i.e., oxidation and/or reduction are crucial for tuning the optical properties of GQDs [23]. So far, an ideal approach to the tunability of the band gap by oxygen functional groups is still a challenge.

In this work, GQDs are chemically doped with Na, K, B, Cl and N (each in solution). The PL and time resolved PL (TRPL) of undoped- doped-GQDs have been extensively studied. It is found that GQDs doped with (Na, K, B) and with (Cl, N) exhibit blue-shift and red-shift of the PL with respect to the undoped GQDs data; corresponding to reduction and oxidation processes, respectively. From the PL results, the average emission energy of our undoped GQDs was determined to be approximately 2.4 eV. The average emission energy is decreased to around 2.3 eV and increased to about 2.8 eV for the most red-shifted and the most blue-shifted samples, respectively. The emission energy tunability of GQDs with only one doping step and a cost effective doping method have been explored and reported for the first time to our knowledge.

## 2. Experimental Details

### 2.1. Preparation Details of Chemically Doped GQDs

**Figure 1:**
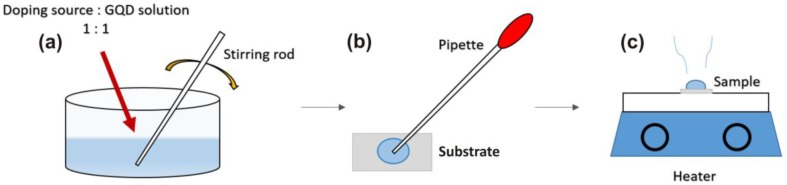
Sample preparation method: (**a**) Graphene quantum dots (GQDs) mixed with reduced dopants in 1:1 amount and stirred; (**b**) Doped GQDs solution drop casted on substrate; and (**c**) substrate heated on hot plate at 85 °C for 10 min.

Water soluble green GQDs (2 mg/mL), purchased from ACS Materials Co. (Medford, OR, USA), were dispersed in a solution containing dimethylformamide (DMF). Doping of GQDs was performed chemically, with dopants in solution. Diluted HCl (hydrochloric acid) was used for Cl-doping, NH_4_OH (ammonium hydroxide) for N-, potassium hydroxide for K-, sodium iodide for Na- and BH_3_O_3_ (boric acid) was used for B-doping, and are represented as Cl-GQDs, N-GQDs, K-GQDS and B-GQDs, respectively. All chemicals were commercially available (Merck, Germany) and used without any further purification. Two different concentrations of each dopant were chosen to compare the effectiveness of doping in GQDs. The doping concentrations of all the solutions were 2 mol/L and 4 mol/L except for boron. The maximum solubility of boron was 2 mol, thus we used 2 mol/L and 1 mol/L solutions to dope the GQDs with B. We will refer to 2 mol/L and 4 mol/L as 2% and 4% respectively, likewise for boron; 1% and 2%, respectively. After reducing each solution, it was mixed with GQDs in 1:1. The solution was stirred for 10 s, the mixed solution was drop-casted on Si substrates followed by heating at 85 °C for 10 min on a hot plate. The Si substrates were post cleaned using RCA, in order to remove all organic and inorganic contamination from them. The preparation procedure is shown in Figure 1. For convenience, Na, K and B doped GQDs are placed in a group named Group A. Likewise, Cl and N are named Group B.

### 2.2. Characterization Techniques

Scanning electron microscopy (SEM) was performed on the undoped GQDs on Si substrate to verify the presence of GQDs on the substrate. The SEM (LEO 1550 Gemini, Zeiss, Germany) was operated at an acceleration voltage of 10 kV. Atomic force microscopy (AFM) in topological and phase mode have been used to study the sample surface morphology and GQDs thickness. AFM was performed using a Dimension 3100 AFM (Digital Instruments, Veeco, Fremont, CA, USA) operated in tapping mode.

For the PL study, the samples were excited with a continuous wave laser with a wavelength of 266 nm (4.66 eV). The spectrum was recorded with a JobinYvon TRIAX 550 spectrometer (Kyoto, Japan) with a focal length of 550 mm, a 600 gr/mm grating, and a liquid nitrogen cooled CCD detector. For the TRPL study, the samples were excited with a 266 nm pulsed laser with a pulse duration of approximately 200 fs and a repetition rate of 75 MHz. The PL transients were recorded by a streak camera from Hamamatsu with a temporal resolution of approximately 10 ps, connected to a spectrometer with a focal length of 500 mm and a 150 gr/mm grating. The TRPL image was recorded by a Peltier cooled CCD.

X-ray photo electron spectroscopy (XPS) measurements were performed in an Axis Ultra DLD instrument (Kratos Analytical, Manchester, UK) using monochromatic Al (Kα) radiation (hv = 1486.6 eV). The base pressure in the analysis chamber during acquisition was <1 × 10^−7^ Pa. XPS core level spectra of the C1s, Cl2p, N1s, B1s and O1s regions, as well as the valance band were recorded. In order to extract the sample composition, a Shirley-type background was subtracted from the core level spectra and elemental cross sections provided by Kratos Analytical were applied. To investigate the bond structure of the GQD, all spectra were referenced to C–C(H) bonds at 285.0 eV. The C1s core level regions were deconvoluted after subtraction of a Shirley-type background using the Casa-XPS software. The C1s core level regions were fitted using Voigt peak shapes with the Lorentzian contribution not exceeding 30%. The full width at half maximum (FWHM) of the components was constrained to 1.8 eV. The number of fitted components in the C1s spectra resembles the chemical composition of the GQD samples owing to the comparatively low differences in binding energy (BE) shifts of C–N, C–Cl and C–O bonds.

## 3. Results and Discussion

**Figure 2:**
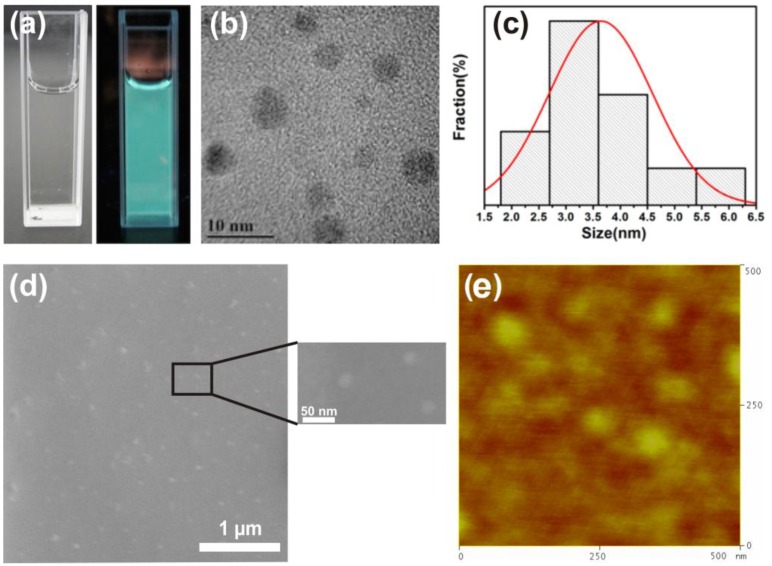
(**a**) GQD dispersed in water under natural light (left) and UV light (right); (**b**) High resolution transmission electron microscopy (TEM) image of GQDs; (**c**) Size distribution of GQDs. (The images (**a**–**c**) are reproduced with permission from [24], Copyright ACS Material, 2016); (**d**) SEM (**e**) Atomic force microscopy (AFM) of GQDs on Si substrate.

Figure 2a displays a photograph of undoped GQDs in aqueous solution, which appears colorless in natural light and exhibits green color in UV light. This confirms the presence of green graphene quantum dots in solution. Figure 2b shows transmission electron microscopy (TEM) images of undoped GQDs and reveal the variable sizes of suspended GQDs in solution. The relative size distribution of GQDs is shown in Figure 2c wherein the maximum particle size is ~6 nm, while the majority of the GQDs show a size ranging between 2.8 nm and 3.6 nm. The undoped and doped GQDs have been dispersed on a Si-substrate for the PL, XPS, SEM and TRPL studies. To confirm the presence of GQDs on the Si substrate and check the spatial distribution of the GQDs, SEM and AFM studies of undoped GQDs were conducted. These studies confirmed the presence of nanoparticles as seen in Figure 2d,e.

Photoluminescence of the GQDs on Si-substrate at room temperature was performed to investigate the luminescence tunability by doping the GQDs. Two groups, the A group (2% and 4% Na- and K-doped GQDs and 1% and 2% of B-GQDs) and the B group (2% and 4% Cl- and N-doped GQDs), were used in this investigation, unless otherwise specified in case of other dopant element were used. We measured the PL in three different positions for each sample with a Si-substrate and found no appreciable variation on different positions. The characteristic peak of undoped GQDs is at 2.4 eV and is consistent with the peak value reported for green GQDs [16,25]. Both blue- and red-shifted PL spectra of the GQDs have been observed, which may be due to an increase or decrease of their band gap, respectively.

**Figure 3:**
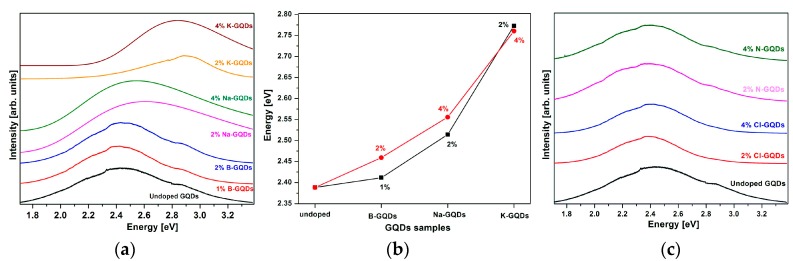
(**a**) Demonstration of blue-shift in photoluminescence (PL) spectra of group A doped GQDs compared to undoped GQDs; (**b**) The change in emission energy as a function of dopant (undoped and group A dopants); (**c**) PL spectra of undoped and group B doped GQDs.

Figure 3a represent PL emission peaks of undoped GQDs and Group A doped GQDs. As seen in Figure 3a, no significant peak shift (average blue-shift of 50 meV) is observed in the PL emission peak for B-GQDs as compared to the undoped GQDs. Zhang et al. reported a blue-shift of B-GQDs, which is consistent with our reported value [26]. Na-GQDs show a larger blue-shift than the B-doped sample, which may be due to intercalation of more Na atoms in the GQDs as compared to B. Furthermore, K-GQDs gives the largest blue-shift, which also may be due to the intercalation of K atoms. Increasing the K concentration from 2% to 4% yields no measureable change in the PL. The average blue-shifts of the emission of GQDs due to Na- and K-doping is about 150 meV and 380 meV, respectively. This has never been reported for Na- and K-doped GQDs before, to our knowledge. In Figure 3b, we present the change in emission energy of the PL as a function of group A dopants. Increasing blue-shift can be correlated with decreasing electronegativity of the dopants involved. Figure 3c shows the PL emission peak of undoped GQDs and Group B (N and Cl) doped GQDs. For this group, no significant peak shifts can be observed in the PL spectra (in average, approximately 60 meV and 40 meV for N-doped and Cl-doped GQDs, respectively).

XPS measurements were performed on the selected samples to observe the change in surface chemistry of GQDs, possibly due to oxygen containing groups. Thus, we selected undoped GQDs, B-doped GQDs from Group A, and Cl- and N- doped GQDs from Group B for XPS measurements.

**Figure 4:**
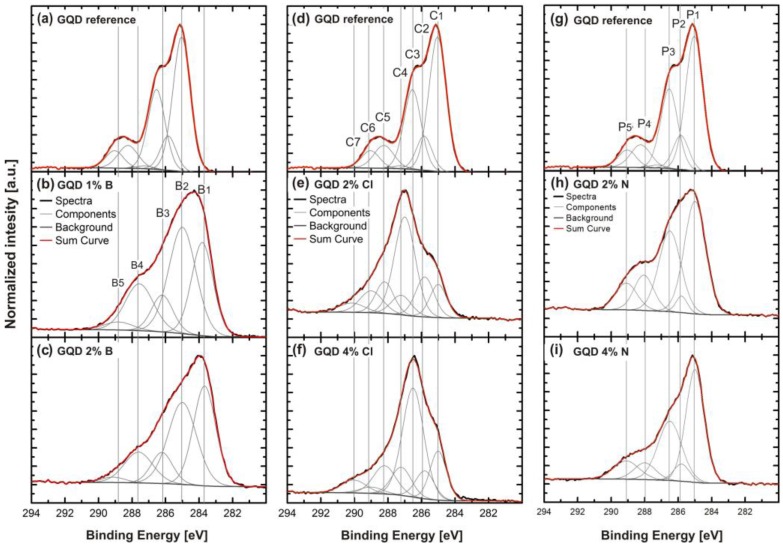
C1s core level spectra of (**a**) the GQD reference sample; (**b**) 1% B-GQDs; (**c**) 2% B-GQDs. The components (B1–B5) and the background are indicated; (**d**) the GQD reference sample; (**e**) 2% Cl-GQDs; (**f**) 4% Cl-GQDs. The components (C1–C7) and the background are indicated; (**g**) the GQD reference sample; (**h**) 2% N-GQDs; (**i**) 4% N-GQDs. The components (P1–P5) and the background are indicated.

Figure 4a–c presents C1s XPS core level spectra of undoped GQDs, 1% and 2% B-doped GDQs. The corresponding C1s core level spectra were deconvoluted into five components referred to as B1-B5. Specifically, the components are assigned to C–B–O bonds at 283.8 eV (B1), C–C/C–CH *sp*^2^ bonds at 285.0 eV(B2), C–C *sp*^3^ at 286.5 eV (B3), C–O bonds at 287.5 eV (B4), and C=O bonds at 289.0 eV (B5) [27,28]. The component B1 (C–B–O) confirms the presence of boron in GQDs which is absent in undoped GQDs and found to increase in concentration as the boron doping increases [26]. The component B2 (C–C *sp*^2^) decreases and B3 (C–C *sp*^3^) increases with increasing B doping. B4 is mainly assigned to C–O bonds and decreases as the doping concentration increases. The B5 assigned to C=O bonding increases with B doping. The reason is discussed in result analysis section.

Figure 4d–f shows C1s core level spectra of undoped GQDs as well as GDQs doped with 2% and 4% Cl. The C1s spectra were deconvoluted into seven components assigned to C–C *sp*^2^ bonds at 285.0 eV (C1), C–C *sp*^3^ bonds at 285.8 eV (C2), C–N bonds at 287.0 eV (C3), C–Cl bonds at 287.2 eV (C4), C–O–N bonds 288.2 eV (C5), C–O/C–O=O at 289.0 eV (C6), and O–C–O=O bonds at 290.2 eV (C7). These values are consistent with the previously reported values [27,28,29,30]. The C3 component resembles C–N bonds as well as C–N bonds influenced by Cl or O. This component shows a 0.5 eV shift towards higher BE for GQDs doped with 2% Cl. Small amounts of N may originate from the preparation procedure of the GQDs. C4, representing C–Cl bonds, shows successful incorporation of Cl atoms in GQDs. C4 increases in concentration as the doping concentration increases. The influence of Cl doping on the oxygen functional groups is visible in component C5 where the increase in oxygen functional groups can be seen as the doping level increases. Likewise, in component C6, a trend of increased oxygen groups can be seen for GQDs doped with 2% Cl but decrease of oxygen functional groups is found as the doping increases to 4% leading to no observable change in the PL of the 2% and 4% Cl-doped GQDs. C7 at 290.1 eV is a component possibly arising from O–C–O=O bonds which is due to attachment of more O-rich groups by doping.

Figure 4g–i presents XPS core level spectra of the C1s region of undoped GQDs and GDQs doped with (2% and 4%) % N. The core level spectra were deconvoluted into a maximum of five components assigned to C–C *sp*^2^ bonds at 285.0 eV (P1), C–C *sp*^3^ bonds at 285.8 eV (P2), C–N bonds at 286.5 eV (P3), C–O–N bonds at 288.0 eV (P4), and C–O/C–O=O bonds at 289.1 eV (P5). These values are consistent with the previously reported values [27,28,31]. The P3 component of C–N is found to decrease in intensity with increasing nitrogen content, which may be due to the fact that an increase in the N concentration results in an increase of the internal pressure in the structure during reactions with C atoms, which removes the less stable N atoms [15,18]. The increase of the FWHM of this peak is observed after increasing the doping concentration of N which may be due to less substitution of N atoms in GQDs but addition of oxygen functional groups as a result of increased doping concentration. The components P4 (C–O–N) and P5 (C–O) are associated with the oxygen functional groups [28]. Here, oxygen functional groups for 2% N-doped are higher as compared to that of 4% N-doped GQDs. Thus, lower N-doping leads to an increase of oxygen containing components similar to Cl-GQDs.

### 3.1. Result Analysis

In GQDs, the *sp*^2^ clusters are embedded in an *sp*^3^ matrix, where *sp*^3^ contains carbon atoms covalently bonded to oxygen atoms (epoxy, hydroxyl groups) and *sp*^2^ are comprised of carbon atoms bonded to carbon atoms or oxygen atoms (carbonyl and carboxyl groups). The energy levels of the *sp*^2^ (π and π*) lie normal to the energy levels of the *sp*^3^ matrix (σ and σ*) and are strongly localized [31,32]. These *sp*^2^ clusters behave as luminescent centers (as being positioned at/or near the Fermi level may be referred to as mid energy gap level) [31] and yield a band gap (π-valence level and π*-conduction level), which is consistent with the observed luminescence (green or blue) [32]. The PL spectrum due to the GQDs is a consequence of recombination of electrons and holes in it. In this scenario, as B-doping in GQDs supports reduction processes (c.f. lower intensities of component B4 and B5, Figure 4b,c). This means that *sp*^2^ clusters are reduced with boron doping (see the lower concentration of *sp*^2^ clusters, i.e., component B2), and hence the mid energy gap level is reduced. In this connection, Robertson et al. explained that the band gap depends inversely upon the size of these clusters. The reduction in the concentration of *sp*^2^ clusters results in a deficiency of electrons in the matrix and hence results in an increase of the band gap of the GQDs [33]. In this study, the sample that shows the largest blue-shift in the PL spectrum has an average emission energy of 2.8 eV. One possible reason for the increase of the emission energy is an increase in the band gap of the GQDs. Luo et al. demonstrated that low reduction leads to removal of C–O (hydroxyl/epoxy) groups with only C=O (carboxyl group) in GQDs, which may explain the observed blue-shift, which is in good agreement to the above discussion for B-GQDs results [21]. This explanation is in accordance with the optical properties of the as-studied GQDs—i.e., the color-tunable emission from green to blue [34]. Likewise, in Cl-GQDs (Figure 4d–f), the concentration of *sp*^2^ clusters (component C1) increases due to slight increase of oxygen containing groups, which in turn reduces the band gap. We correlate this observation with the red-shift observed in the PL spectra due to Cl-doping of the GQDs. This is in agreement with the previous work by Yeh et al. and Luo et al. [19,21]. Moreover, in N-doped GQDs) we notice that the concentration of oxygen functional groups increases (cf. Figure 4g–i component P4 and P5). This increases the concentration of *sp*^2^ clusters in the GQDs (component P1). As a result, a mid energy gap level is created and we argue that this might be the reason for the reduction (down to an average of 2.3 eV) of the emission energy of the N-doped GQDs. Furthermore, it has previously been shown that the oxidation behavior (electron donating behavior) of group B dopants (Cl and N) leads to an increase in the electron density in GQDs [35,36].

**Figure 5:**
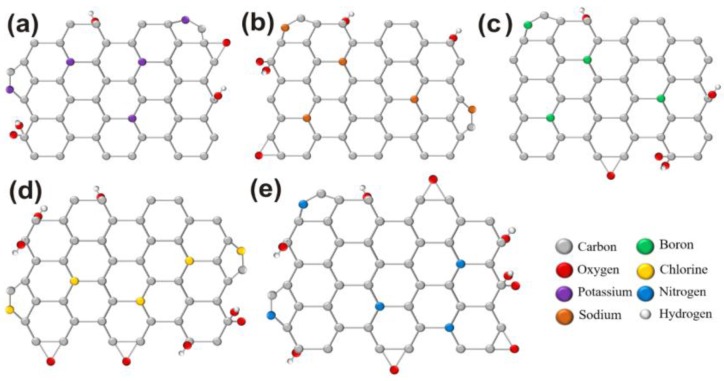
Schematic illustration for (**a**) K-GQDs; (**b**) Na-GQDs; (**c**) B-GQDs; (**d**) Cl-GQDs; and (**e**) N-GQDs showing the change in oxygen groups as a result of K, Na, B, Cl and N doping.

Figure 5a–c shows the possible crystal structures of K-, Na-, and B- doping and respectively, the possible changes in oxygen groups. Figure 5d,e shows the possible structures with the involvement of oxygen groups for Cl and N doped GQDs, respectively.

### 3.2. Time Resolved Photoluminescence

**Figure 6:**
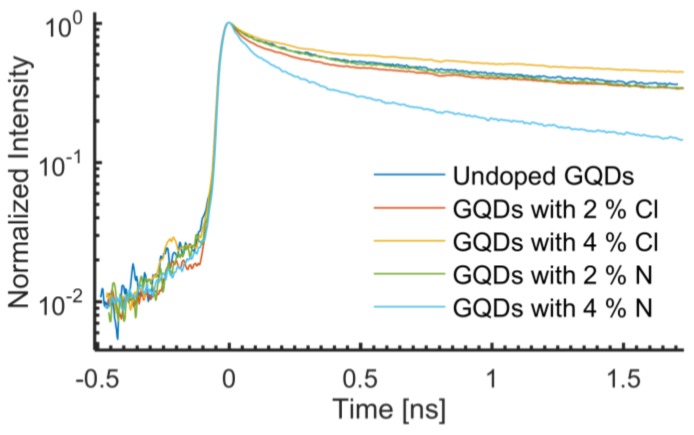
PL transients of undoped and 2% and 4% Cl- and N-doped GQDs on Si.

A time resolved photoluminescence study of Cl- and N-doped GQDs was purposefully conducted at room temperature. Each PL transient analyzed consists of a spectral integration of the temporal PL data from 2.62 eV to 2.33 eV. Figure 6 shows a collection of PL transients for the undoped sample and the 2% and 4% Cl and N doped GQDs. The non-exponential decays of the transients have been fitted with the following bi-exponential function:

$$I=\alpha_{1}e^{-\frac{t}{\tau_{1}}}+\alpha_{2}e^{-\frac{t}{\tau_{2}}}$$

The parameters α_1_ and α_2_ are proportionality constants, and *t* is the time. With this simplified model, the PL is explained as having two lifetime components (one short, τ_1_, and one long, τ_2_). The undoped sample, the Cl doped samples, and the 2% N doped sample do not show significant differences in the PL lifetimes. They exhibit short lifetime components, τ_1_, ranging from 0.13 ns to 0.15 ns, and long lifetime components, τ_2_, ranges from 2.9 ns to 3.7 ns (see Table 1). Normalizing the proportionality constants to add up to 100% for each fit yields a weight of approximately 40% for the quicker recombination channel and approximately 60% for the slower recombination channel of the aforementioned four samples (see Table 1).

**Figure 7:**
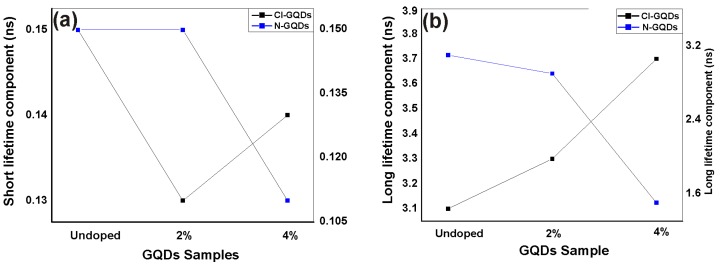
(**a**) Trend of short lifetime component of undoped Cl-GQDs and N-GQDs; (**b**) Trend of long lifetime component of undoped Cl-GQDs and N-GQDs.

In view of excitation and de-excitation processes of charge carriers, different phenomena have been observed for N- and Cl-doping. The emission from undoped GQDs originates from intrinsic states of *sp*^2^ clusters present in them [16]. As proposed earlier, the effective doping of N and Cl, generates mid-gap energy levels together with additional oxygen functional groups in the GQDs, which is in agreement with the literature [37]. A qualitative understanding of this phenomenon is as follows: after absorbing an incident (laser) photon, an electron is excited from HOMO (highest occupied molecular orbital) to LUMO (lowest unoccupied molecular orbital) of the excited states of oxygen groups. After a characteristic lifetime, the electron relaxes back down to HOMO by sending out a photon. In this work, we refer to this transition energy as the band gap. During relaxation, the short lifetime component showed slight change (almost negligible) for undoped N and Cl doped GQDs as shown in Figure 7a. The short component of the PL lifetime is assumed to originate from excitons at intrinsic states that quickly disappear in initially populated core states due to repulsion from other electrons and holes or may be due to quick recombination of electron holes in the populated intrinsic states [38].

The 2% N-doped sample showed very similar transients as those observed for the undoped GQDs, with short and long lifetime components of 0.15 ns and 2.9 ns, respectively. Increasing the N concentration to 4% resulted in a significant reduction in the effective PL lifetime, yielding a short and long lifetime component of only 0.11 ns and 1.5 ns, respectively. More notably, the 4% N-doped sample has a considerably stronger contribution from the quicker recombination channel, as compared with the other four samples. The weight of the short lifetime component is 60%, compared to about 40% for the other samples. The stronger contribution from the quicker recombination channel leads to shorter observed lifetime components, as only the first part of the transients could be observed in our 2.2 ns time frame.

Unlike Cl-GQDs, the oxygen functional groups in N-GQDs provide different non-radiative channels which affect the lifetime and may be the reason of decrease in long lifetime components observed with N-doping as is clear from Figure 7b. Deng et al. demonstrated that in N-GQDs, the oxygen functional groups induced trapping centers between HOMO and LUMO which quench the luminescence and is the reason of observed red-shift in PL [39]. Therefore, it is believed that the quenching is due to an increase in non-radiative channels by oxygen functional groups which act as trapping centers and is the reason of reduced long lifetime components [3,40].

## 4. Conclusions

In summary, we have developed a one-step and low-cost method for emission energy tuning of GQDs using different dopants in solution form. The dopants used for doping in GQDs are potassium, sodium, and boron (Group I–IV) named group A dopants, whereas nitrogen and chlorine (Group V–VIII) are named group B dopants. The concentration of the oxygen functional groups are inversely proportional to the band gap and hence reduce their concentration results in an increase of the emission energy. The XPS study of group A doped GQDs samples shows a reduction and/or inactiveness of the oxygen functional groups. Doping of GQDs with group A (K, Na, and B) results in a blue-shift of the emission energy of up to 0.4 eV. Likewise, doping of GQDs with group B (Cl and N) dopants result in a red-shift of the PL. The XPS study of group B doped GQDs samples shows an increase of the concentration of the oxygen functional groups, as compared to the undoped GQDs. This increase in the concentration of the oxygen functional groups results in a red-shift of the emission energy of 0.1 eV. The PL and XPS observations are in good agreement with each other. Moreover, the TRPL study of GQD samples with different concentrations of Cl and N indicates that there is more than one recombination channel for the charge carriers. Cl substitution in GQDs has no significant effect on the recombination rate. Doping with 4% of N, however, leads to an increase of the recombination rate, due to an increased contribution from the fast recombination channel, which may be non-radiative. In conclusion, chemically doped-GQDs can be used for making low cost solution based photodetectors, green and blue LEDs, as well as solar cells.

## Figures and Tables

**Table 1:** The short and long lifetime components of N- and Cl-doped GQDs samples.

Samples	$\alpha_{1}$ (%)	$\tau_{1}$ (ns)	$\alpha_{2}$ (%)	$\tau_{2}$ (ns)
GQDs	40	0.15	60	3.1
2% Cl-GQDs	43	0.13	57	3.3
4% Cl-GQDs	39	0.14	61	3.7
2% N-GQDs	42	0.15	58	2.9
4% N-GQDs	60	0.11	40	1.5
